# Primary melanoma of the cornea

**DOI:** 10.3205/oc000139

**Published:** 2020-03-18

**Authors:** Dimitrios Z. Panagiotou, Angeliki A. Chranioti, Sofia-Eleni Tzorakoleftheraki, Nikolaos G. Ziakas, Panagiotis K. Oikonomidis

**Affiliations:** 1 1st Department of Ophthalmology, Aristotle University of Thessaloniki, AHEPA Hospital, Thessaloniki, Greece; 2 Department of Ophthalmology, Brugmann University Hospital, Brussels, Belgium; 3 Department of Ophthalmology, General Hospital of Karditsa, Greece; 4 Department of Pathology, Aristotle University of Thessaloniki, AHEPA Hospital, Thessaloniki, Greece

**Keywords:** corneal melanoma, cornea, topical chemotherapy

## Abstract

**Purpose:** To present an extremely rare case of corneal melanoma.

**Method:** An 84-year-old female patient presented to our department with a pigmented corneal lesion in her right eye (OD), 6x4 mm, complaining of mild pain and inability of complete eyelid closure. Tumor growth had been noted the previous year. She had undergone cataract surgery in her right eye three years before, followed by an unspecified postoperative complication. Her visual acuity was 3/10 OD and 9/10 OS. Ophthalmic evaluation and ultrasonography (A- and B-scan) did not reveal any other pathology. The pigmented lesion was surgically removed and the patient underwent a protocol therapy of topical chemotherapy (mitomycin 0.03%, 2x4 for 2 weeks and dexamethasone 0.1%, 2x4 for the following 2 weeks, followed by another cycle of mitomycin 0.03%, 2x4 for another 2 weeks).

**Results:** The surgical removal of the lesion was uncomplicated, as was the postoperative period. The patient’s visual acuity improved to 6/10 three months postoperatively. The histologic examination revealed malignant melanoma.

**Conclusions:** Despite its rarity, primary melanoma of the cornea is an existing entity. Treatment of corneal melanoma consists of surgical removal and postoperative topical chemotherapy. Postoperative follow-up is mandatory.

## Introduction

Primary melanoma of the cornea is an extremely rare clinical entity, which clearly differs from conjunctival melanoma extending onto the cornea. The first case of corneal melanoma was reported in 1892 [1] and since then there have only been eighteen such case reports in the international (medical) literature to date [2], [3], [4], [5], [6], [7], [8], [9], [10], [11], [12], [13], [14], [15], [16]. Furthermore, the research documenting its origin remains relatively weak. Given the rarity of the disease in combination with our motivation to contribute as much as possible to its investigation, we present the following case report.

## Case description

**Figure 1:**
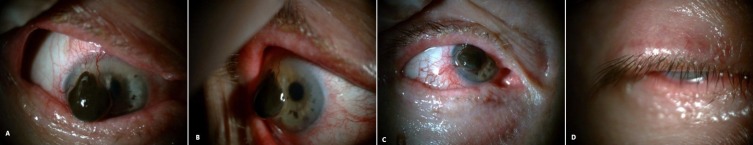
Primary melanoma of the cornea; A), B) and C) corneal pigmented lesion, clearly demarcated and surrounded by clear cornea, with two feeder vessels derived from the limbus; D) inability of complete eyelid closure

In December 2015, an 84-year-old woman presented to the 1st Department of Ophthalmology, Aristotle University of Thessaloniki, AHEPA Hospital, Greece, complaining of gradual vision deterioration, pain, and inability of complete eyelid closure (Figure 1 D (Fig. 1)) of her right eye (OD). Her ophthalmic history revealed cataract surgery OD three years before, followed by an unspecified postoperative complication. Moreover, a pigmented corneal lesion had been noted in the same eye one year earlier, which exhibited constantly increasing dimensions. Her general medical history revealed arterial hypertension, adrenal adenoma, hiatus hernia, and right facial nerve paresis for the past twenty-five years.

Best corrected visual acuity (BCVA) was 3/10 OD, with the head tilted to the right and 9/10 OS. Slit-lamp examination of her right eye revealed a circular corneal pigmented lesion, measuring 4x6 mm, clearly demarcated and surrounded by clear cornea of over 1 mm. Two feeder vessels passed through the limbus onto the lesion (Figure 1A-C (Fig. 1)). The presence of the lesion did not allow IOP measurement, gonioscopy or fundoscopy. Ophthalmic assessment of the left eye did not reveal any pathology other than an incipient clouding of the crystalline lens.

**Figure 2:**
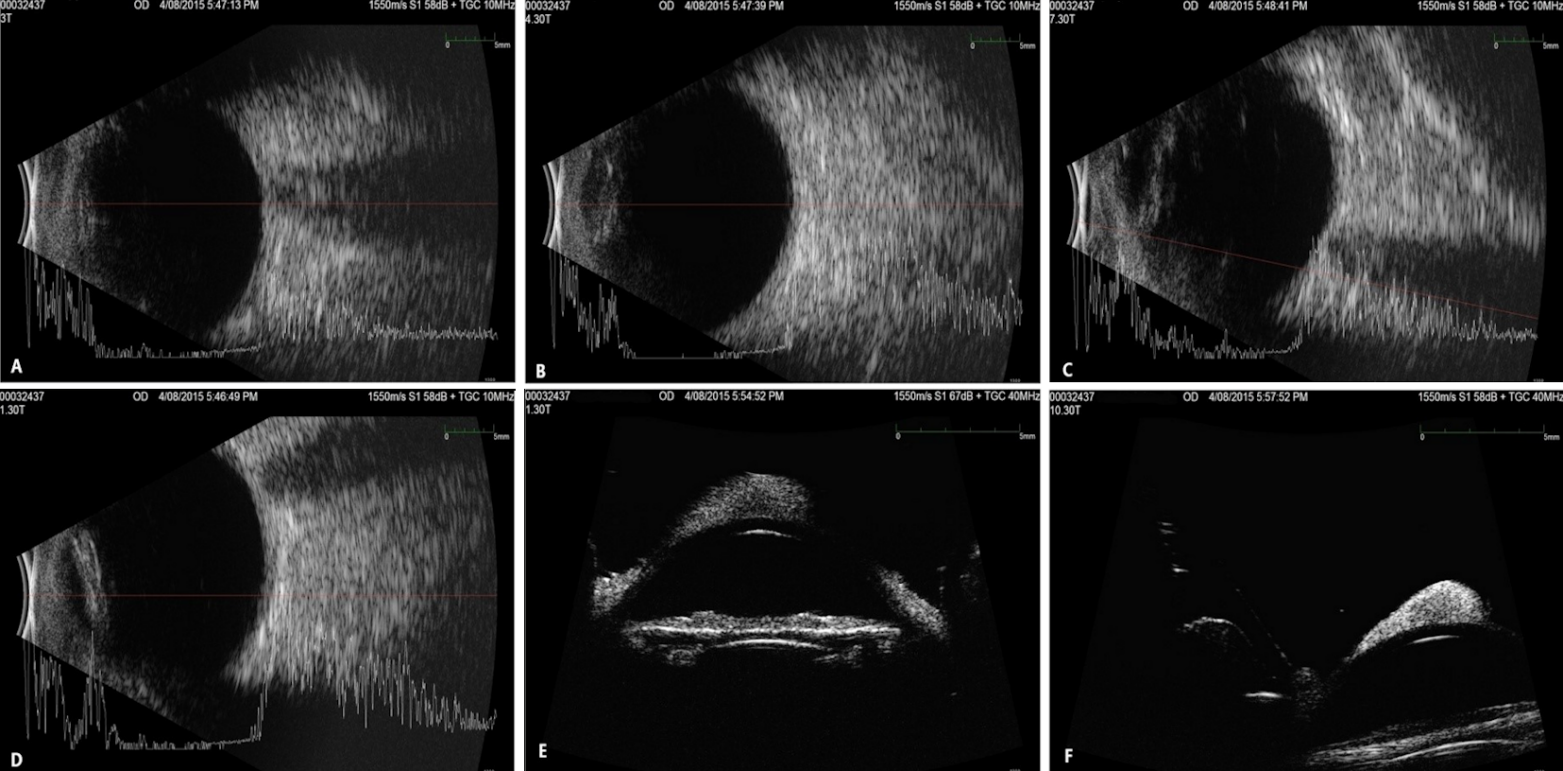
Imaging which did not reveal associated pathology; A), B), C) and D) A- and B-scan; E) and F) UBM – lesion sparing posterior stroma and endothelium; (courtesy of Dr. Miltos Balidis, OPTHALMICA Eye Institute)

**Figure 3:**
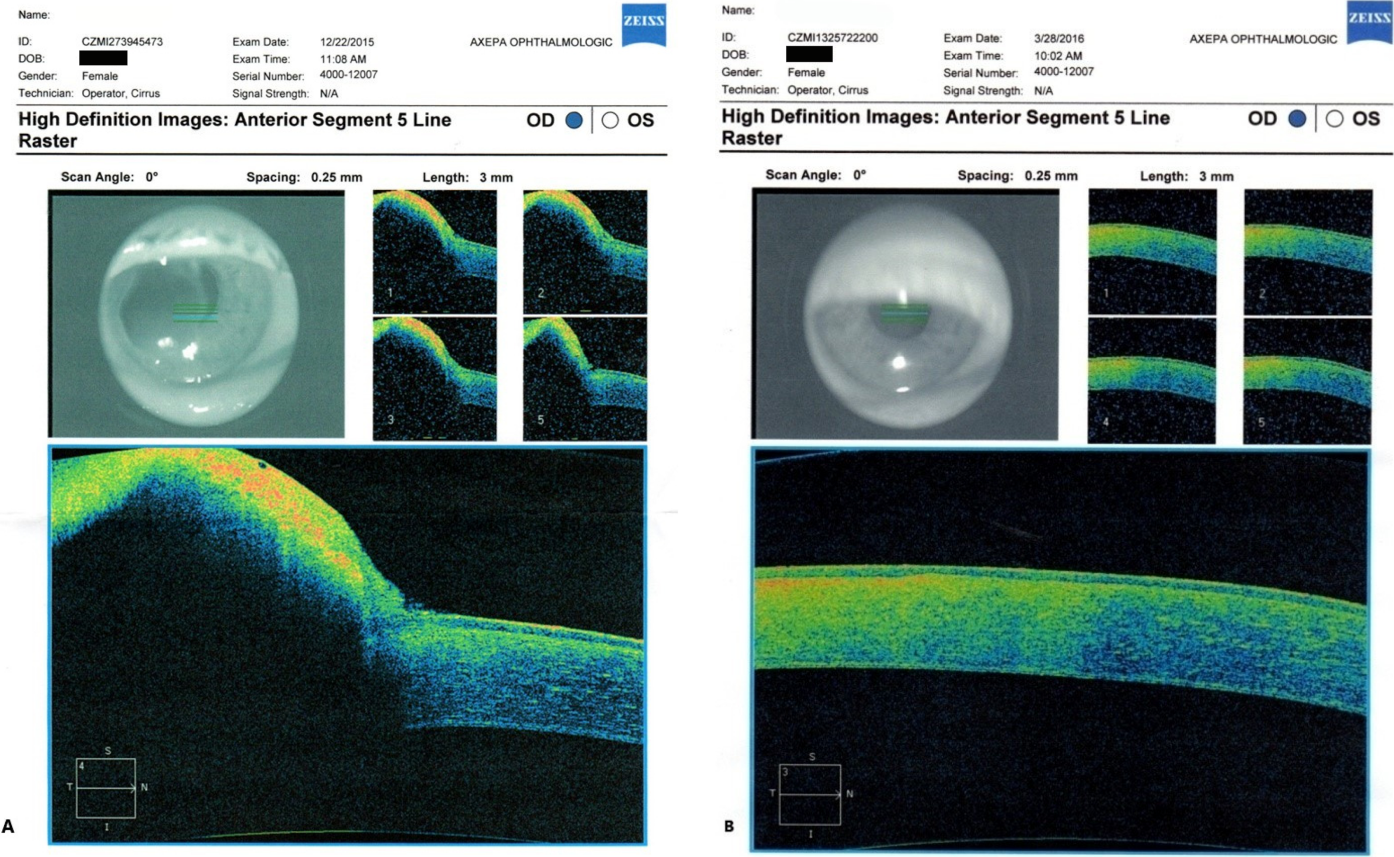
AS-OCT; A) preoperative; B) post-operative

The patient underwent ultrasonography (A- and B-scan, Figure 2A-D (Fig. 2)), ultrasound Biomicroscopy (UBM, Figure 2E-F (Fig. 2)) and anterior segment OCT (AS OCT, Figure 3A (Fig. 3)), which did not reveal associated pathology, as did required examinations according to clinic protocol for patients with newly diagnosed ocular tumor.

### The required examinations according to clinic protocol

- CBC
- Liver function tests, ALP, LDH, K, Na, Ca
- Chest X-ray
- MRI brain – orbits
- U/S (upper abdomen and pelvis)
- Bone scan

Differential diagnosis included tumors of the ocular surface, with corneal melanoma being the most prevalent based on strong clinical suspicion and published data regarding tumor atypia [17], [18].

### Differential diagnosis

- Papilloma
- Naevus
- Squamous cell carcinoma
- Primary acquired melanosis (PAM)
- Malignant melanoma

### “Five rules of atypia”

1. Irregular tumor boundaries
2. Change and/or color, shape, size variation
3. Presence of epithelial defects or ulcers on the surface
4. Dilated feeder blood vessels leading to the tumor
5. Displacement and/or infiltration of adjacent tissues

**Figure 4:**
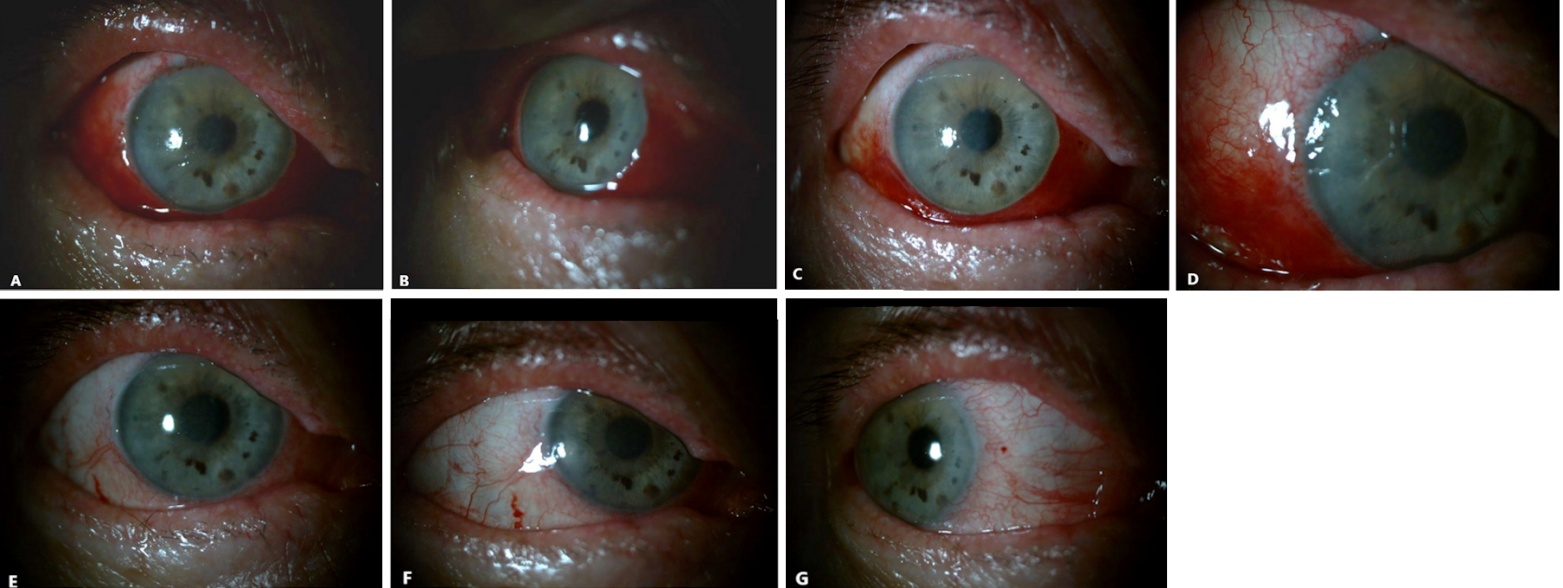
Photographs taken post-operatively; A) and B) day 1; C) and D) week 6; E), F) and G) week 8

Diagnostic and therapeutic management included surgical excision. Following cauterization of the feeder vessels, the lesion was removed on healthy boundaries via excision and scraping of the corneal epithelium [19]. The patient underwent topical chemotherapy (mitomycin 0.03%, 2x4 for 2 weeks and dexamethasone 0.1%, 2x4 for the following 2 weeks, followed by another cycle of mitomycin 0.03%, 2x4 for another 2 weeks), while awaiting the histopathologic analysis [15], [18], [19]. An uncomplicated postoperative period followed, as is documented in the AS OCT (Figure 3B (Fig. 3)) and photographs taken on postoperative day 1 (Figure 4A and B (Fig. 4)), week 6 (Figure 4C and D (Fig. 4)) and week 8 (Figure 4E-G (Fig. 4)). Best corrected visual acuity reached 6/10. Twenty months of follow-up later, the postoperative result is cosmetically and functionally excellent, with no evident signs of local or distant recurrence.

**Figure 5:**
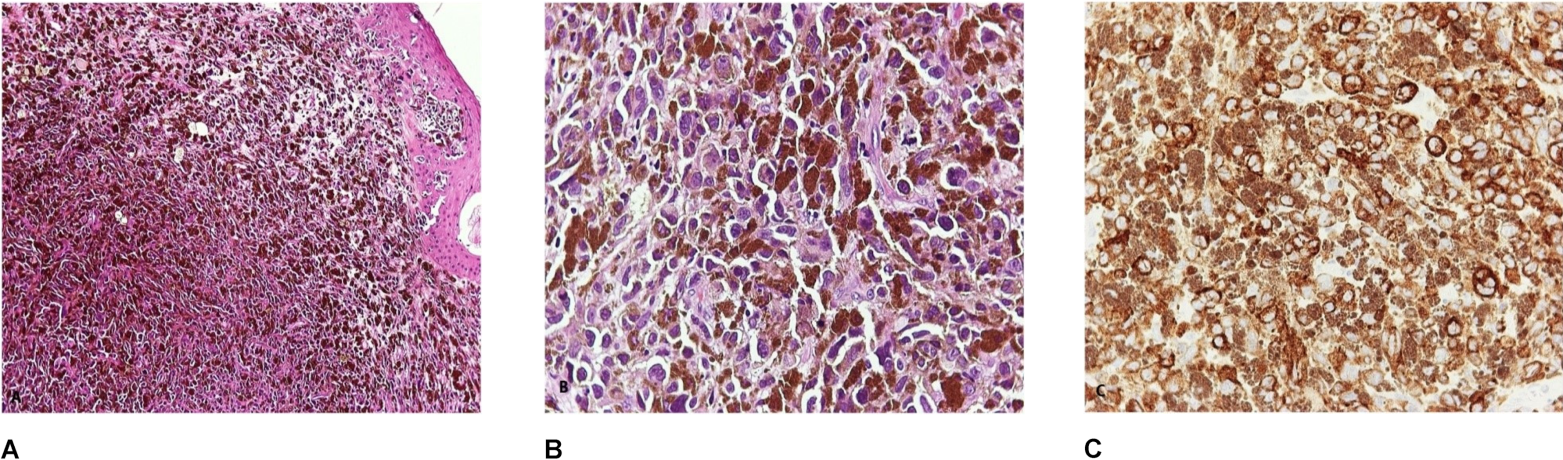
Histologic features; A) low-power view of the neoplasm, hematoxylin-eosin, x100; B) high-power view illustrating the cytologic features of the tumor cells, hematoxylin-eosin, x400; C) positive immunostaining of the tumor cells for Melan-A antigen, automated immunohistochemistry, x400; (courtesy of Prof. Prodromos Chitiroglou, Pathology Department, Aristotle University of Thessaloniki, Greece)

Histopathologic analysis confirmed our clinical suspicion. The corneal lesion specimen examined by the Pathology Department measured 0.5x0.4x0.3 cm and had gray-black colour. Hematoxylin- and eosin-stained sections revealed malignant corneal tissue involving the junction of the epithelium and the lamina propria, as well as the underlying connective tissue in its full thickness (Figure 5A (Fig. 5)). The neoplasm consisted of large-sized, round, polygonal, and spindle cells, with eosinophilic cytoplasm and hyperchromatic, markedly pleomorphic nuclei, with large nucleoli (Figure 5B (Fig. 5)). The tumor cells contained numerous cytoplasmic melanin granules (Figure 5A and B (Fig. 5)). Many multinucleated neoplastic cells were observed. Neoplastic cell arrangement included confluent clusters, islets or single cells, invading connective tissue and the overlying stratified squamous epithelium. Mitotic activity was mild. Immunohistochemical stains revealed tumor cell diffuse positivity for HMB45 and Melan-A antigens (Figure 5C (Fig. 5)). Stain for Ki-67 proliferation antigen showed positive neoplastic cells throughout the tissue fragment. Based on the aforementioned findings, diagnosis of malignant melanoma of the cornea was made.

## Discussion

As initially stated, this report refers to an extremely rare occurrence. Corneal involvement of a melanotic lesion of the eye is not rare; but melanoma strictly involving the cornea along with the absence of any damage to the rest of the eye’s structures has actually been a rather infrequent report in the international literature. This could explain the insufficient documentation of the disease’s pathogenesis.

Further study is required regarding the etiology of the melanocytes’ presence in the cornea and the conditions under which differentiation takes place. Nevertheless, some theories have been formulated based on research data and small clinical experience, the most prevalent of which advocates that healthy corneal tissue does not have any melanocytes. In certain cases, however, melanocytes migrate from the limbus to the corneal epithelium under the effect of solar radiation or chemical substances [13], [20]. This migration in combination with immersion into the corneal stroma is facilitated by previous injury to Bowman’s membrane due to a labor-intensive, complicated surgery, or a penetrating trauma [12], [14], [16], [20], [21], [22], [23]. Bowman’s membrane cannot regenerate, thus the migration of melanocytes is insufficiently hindered [20]. Immigration per se signals the effort of limbus cells to restore the integrity of the cornea layers [21]. This theory is also reinforced by the observation that migration occurs parallel to vascularization; both seem to have the same stimulus, probably under an inflammatory response [16], [21], [22], [23]. The clear delineation of the lesion and its feeder vessels in a triangular area of the cornea impedes metastases and therefore yields a good prognosis [20], [21].

Some authors have suggested that corneal lack of melanin supervenes due to the failure of corneal melanocytes to synthesize melanin and consider basal cells of the corneal epithelium as potential melanoblasts [23]. Others report the presence of melanin in Hudson-Stahli lines and in Schwann cells of corneal nerves [8]. This report aims to provide impetus for further research regarding primary corneal melanoma.

## Conclusions

In conclusion, primary melanoma of the cornea is an extremely rare ocular tumor whose pathogenesis necessitates further study. Treatment consists of surgical resection, with or without cryotherapy, biopsy of the resected lesion and postoperative topical chemotherapy. Absence of metastases provides ground for good prognosis.

## Notes

### Competing interests

The authors declare that they have no competing interests.

### Acknowledgements

The case has been presented at the XXXIV Congress of the European Society of Cataract and Refractive Surgeons (ESCRS), September 2016, Copenhagen, Denmark.

Dr. Panagiotou received a bursary from the ESCRS for attending the Congress and presenting this case.

We express sincere thanks to Prof. Leonidas Zografos for his advisory guidance.

A- & B-scan and UBM images courtesy of Dr. Miltos Balidis.

Histologic images courtesy of Prof. Prodromos Chitiroglou.
